# Modular-Based Synergetic Mechanisms of Jasminoidin and Ursodeoxycholic Acid in Cerebral Ischemia Therapy

**DOI:** 10.3390/biomedicines13040938

**Published:** 2025-04-11

**Authors:** Jingai Wang, Qikai Niu, Yanan Yu, Jun Liu, Siqi Zhang, Wenjing Zong, Siwei Tian, Zhong Wang, Bing Li

**Affiliations:** 1 Institute of Chinese Materia Medica, China Academy of Chinese Medical Sciences, Beijing 100700, China; wangjingai928@163.com (J.W.); zhangsq0223@163.com (S.Z.); caoyeying.student@sina.com (W.Z.);; 2 Institute of Basic Research in Clinical Medicine, China Academy of Chinese Medical Sciences, Beijing 100700, China; pumpkinnaicha@163.com (Y.Y.); franlj1104@aliyun.com (J.L.)

**Keywords:** combination therapy, synergistic effect, network topology, modular analysis, cerebral ischemia

## Abstract

**Objectives**: Jasminoidin (JA) and ursodeoxycholic acid (UA) have been shown to exert synergistic effects on cerebral ischemia (CI) therapy, but the underlying mechanisms remain to be elucidated. Objective: To elucidate the synergistic mechanisms involved in the combined use of JA and UA (JU) for CI therapy using a driver-induced modular screening (DiMS) strategy. **Methods**: Network proximity and topology-based approaches were used to identify synergistic modules and driver genes from an anti-ischemic microarray dataset (ArrayExpress, E-TABM-662). A middle cerebral artery occlusion/reperfusion (MCAO/R) model was established in 30 Sprague Dawley rats, divided into sham, vehicle, JA (25 mg/mL), UA (7 mg/mL), and JU (JA:UA = 1:1) groups. After 90 minutes of ischemia, infarct volume and neurological deficit scores were evaluated. Western blotting was performed 24 h after administration to validate key protein changes. **Results**: Six, eleven, and four drug-responsive On_modules were identified for JA, UA, and JU, respectively. Three synergistic modules (Sy-modules, JU-Mod-7, 8, and 10) and 12 driver genes (e.g., NRF1, FN1, CUL3) were identified, mainly involving the PI3K-Akt and MAPK pathways and regulation of the actin cytoskeleton. JA and UA synergistically reduced infarct volume and neurological deficit score (2.5, *p* < 0.05) in MCAO/R rats. In vivo studies demonstrated that JU suppressed the expression of CUL3, FN1, and ITGA4, while it increased that of NRF1. **Conclusions**: JU acts synergistically on CI–reperfusion injury by regulating FN1, CUL3, ITGA4, and NRF1 and inducing the PI3K-Akt, MAPK, and actin cytoskeleton pathways. DiMS provides a new approach to uncover mechanisms of combination therapies.

## 1. Introduction

Cerebral ischemic stroke is a common cause of death and disability. It has high incidence, disability, and mortality rates [1]. It is a major problem that threatens human health and quality of life. Current treatments primarily include thrombolytic therapy, such as recombinant tissue plasminogen activator (rt-PA) [2], endovascular thrombectomy [3,4], anticoagulant/antiplatelet therapy [5], and neuroprotective agents [6,7,8]. Increasing evidence shows that combination therapy is more advantageous for ischemic stroke since it improves the therapeutic effect without increasing side effects [9,10]. An in-depth study of ischemic stroke showed that herbal combination therapy is an effective treatment for cerebral ischemia (CI) [11,12]; however, the mechanisms driving the synergistic effects of combination therapy remain unclear. It is crucial to understand the synergistic mechanisms of combination therapy, which could provide valuable insights and improve the treatment of cerebral ischemia. In recent years, several computational approaches were developed to identify combination therapy targets. This includes co-expression networks based on modular analysis that are based on association genes or proteins and allow a deeper understanding of the underlying mechanisms of drug use in complex diseases [13,14,15].

Ischemic stroke triggers complex pathological processes, including excitotoxicity [16], oxidative stress [17], and inflammation [18], leading to neuronal damage and functional deficits [19]. Single-target therapies often struggle to effectively control the multiple mechanisms of injury associated with stroke [20]. Therefore, multi-target synergistic treatment strategies have gained increasing attention. Qingkailing injection, a widely used herbal compound in stroke treatment, contains two main bioactive components: jasminoidin (JA) and ursodeoxycholic acid (UA). JA, derived from the traditional Chinese medicine Fructus Gardeniae, has shown potential in addressing oxidative stress, inflammation, and neuronal apoptosis [21]. UA, derived from calculus bovis factitius, exhibits a range of pharmacological effects, including immunosuppression, inhibition of calcium influx, prevention of neurodegeneration, strong anti-inflammatory activity, and neuroprotection through mitochondrial stabilization and apoptosis inhibition [22,23]. Unlike arbitrary combinations of antioxidants and anti-inflammatory agents, *Fructus Gardeniae* and artificial bezoars have been historically co-administered in traditional formulations, with their active ingredients, JA and UA, suggesting the possibility of synergistic effects. However, studies investigating the synergistic effects of JA and UA remain limited, with a lack of in-depth mechanistic analysis. Given the multi-faceted nature of stroke pathology, it is crucial to explore the combined effects of JA and UA. While single-target therapies often fail to fully address the complex biological processes of stroke, JA and UA may offer broader neuroprotection by targeting multiple signaling pathways simultaneously. Although their individual efficacy has been demonstrated, the mechanisms underlying their combined therapy are still under-explored. This research aims to fill this gap by using a modular-pharmacology-based approach to explore the combined effects of JA and UA, providing insights into their synergistic mechanisms.

Despite the promise of combination therapies, existing studies often lack a deep mechanistic understanding of the synergies involved. This study seeks to address this gap by using a network topology approach to identify key synergistic modules and driver genes. Network topology has become an essential tool in pharmacology for decoding disease mechanisms, accelerating drug discovery, and repositioning existing drugs. For example, the BrainMI framework integrates brain functional connectivity with molecular genetic networks to identify genes involved in neurological disorders [24]. Network topology methods have been successfully applied in antiviral drug repositioning [25], drug-target prediction [26], and traditional Chinese medicine [27]. By employing this network-driven approach (network proximity, $S_{AB}$), network pharmacology reveals complex molecular interactions that drive therapeutic synergies [28].

This study used module-based separation measure of $S_{AB}$ (network proximity of drugs A and B) and network topology methods to identify JU synergistic modules and driver genes based on the anti-ischemic genome network. The driver-induced modular screening (DiMS) strategy was used to elucidate the synergistic mechanism of JU in CI treatment through further functional analysis and literature verification.

## 2. Materials and Methods

### 2.1. Gene Expression Dataset

The microarray gene expression dataset containing 16,463 mouse cDNAs (Inocyte Genomics, Inc., Santa Clara, CA, USA) was constructed in our previous study and was uploaded to the Array Express database: http://www.ebi.ac.uk/arrayexpress,E-TABM-662 (accessed on 19 August 2022) [29]. Sham, JA (25 mg /mL), UA (7 mg/mL), and JU (a combination of jasminoidin and ursodeoxycholic acid at a ratio of 1:1) were selected for analysis.

Experimental Design: Healthy adult male Kunming mice (12 weeks, 38–48 g) were used in this study. These mice were free of specific pathogens and were housed at 25 °C under a 12 h light/dark cycle. A model of middle cerebral artery occlusion (MCAO) was surgically established as described in our previous studies [29,30]. Specifically, the left middle cerebral artery was blocked using intracavitary filaments for 1.5 h, followed by 24 h of reperfusion to induce ischemia–reperfusion. Histological analysis and cerebral infarction area measurements were then performed, as detailed in the cited references.

### 2.2. Establishing and Identifying of Co-Expression Modules

Weighted gene co-expression network analysis (WGCNA) was performed for gene co-expression network construction and module detection based on data from the single drug group (JA and UA) and combined drug group (JU) in the above database using R software (version 4.3.2) [31]. The topological overlap matrix (TOM) is defined as the similarity of each matrix between all probe pairs in the measurement sample. Hierarchical clustering and a dynamic hybrid tree-cutting algorithm were applied to detect the tree branch. The tree branch was divided into modules, and the appropriate soft threshold (the ordinal) was selected. The gray color represents genes that were not classified into any module [32]. The scale-free topology fit index (SFT.R.sq) reaches or approaches 0.8, indicating that the network exhibits scale-free network characteristics and can effectively reflect gene–gene interactions and regulatory relationships. Therefore, we selected soft threshold values (β) that resulted in an SFT.R.sq value of 0.8 or close to 0.8. In this study, the β values were as follows: β = 10 (for JA), β = 16 (for UA), and β = 20 (for JU). Based on these β values, we constructed the pairwise correlation matrix of all probes within the samples, with a minimum module size of three for all three groups.

### 2.3. Identification of Drug Response On_Module and Sy-Module

The $Z_{summary}$ statistic was implemented in the module conservation function of WGCNA to quantitatively determine whether the co-expression pattern of modules in the drug set was vector-independent. This assesses whether the density and connectivity patterns of modules defined in the reference dataset were present in the test dataset [33]. The $Z_{summary}$ statistic, implemented in the module preservation function of WGCNA, is used to quantitatively assess whether the co-expression pattern of gene modules in a drug treatment dataset is consistent with that of a reference dataset [33]. $Z_{summary}$ evaluates module preservation by considering both module density and gene connectivity patterns. It is composed of four statistics related to module density and three related to gene connectivity. A high $Z_{summary}$ value (≥2) indicates good module preservation, meaning that gene co-expression relationships remain stable across datasets. Conversely, a low $Z_{summary}$ value (<0) suggests poor module preservation, with $Z_{summary}<0$ typically indicating structural instability influenced by experimental conditions, such as drug treatment, which may alter gene co-expression patterns. These unstable modules often contain key regulatory genes, making them valuable for investigating drug mechanisms or regulatory pathways involved in biological responses to external interventions.

In this study, we classified modules into two categories based on their $Z_{summary}$ values:

On_module ($Z_{summary}<0$): Gene modules that exhibit significant changes under drug treatment, likely activated or substantially regulated by the drug. These modules represent biological processes influenced by the drug and reflect its biological effects through the activation of specific gene modules. On_modules are considered to be potential targets or pathways that could be modulated by the drug treatment, making them crucial for understanding the pharmacological mechanisms involved.

Off_module ($Z_{summary}\geq 2$): Gene modules that are not significantly affected by drug treatment and may represent biological processes or pathways not regulated by the drug. Their low response to the drug suggests they may maintain homeostasis within the body. Off_modules help distinguish the biological processes that remain stable in response to treatment, offering a clearer understanding of the drug’s specific action [15].

The formula to calculate $Z_{summary}$ is as follows [33]:

$$Z_{summary}=\frac{median(Z_{mean\;Cor,\;ZmeanAD},\;Z_{propvarExpl},\;Z_{meamKME})+median(Z_{cor.KIM},\;Z_{cor,\;KME},\;Z_{cor.cor})}{2}$$

Similarly, JA and UA were used as control groups for comparison with JU, respectively, and the modules obtained by JU were regarded as synergetic modules (Sy_modules).

### 2.4. Analysis of Differentially Expressed Genes (DEGs)

Drug-induced DEGs were screened using limma in R based on data from the sham, JA, UA, and JU groups [34]. The DEGs in the administration and sham operation groups were screened using a fold change (FC) > 1.2 and *p* < 0.05 as the threshold, and Bonferroni correction was performed to screen the list of DEGs in different administration groups for further analysis.

### 2.5. Synergistic Prediction Analysis

We need to clarify whether the topological relationship between the target modules of the two drug sets reflects the link between biology and pharmacology and verify the effectiveness of the network-based drug combination approach using the relevant modules obtained above. $S_{AB}$ is a metric that measures the distribution of a drug target set within a human protein–protein interaction (PPI) network and is used to assess whether a drug combination exhibits potential synergistic effects. We similarly applied this metric to determine the relationship between modules. The following method calculates $S_{AB}$ by measuring the ’separation’ between drug groups A and B to validate the rationale for combining JA and UA [25].

$$S_{AB}=\langle d_{AB}\rangle -\frac{\langle d_{AA}\rangle +\langle d_{BB}\rangle }{2}$$

where $\langle d\rangle$ is calculated according to the ‘closest’ method, $\langle d_{AA}\rangle$ represents the shortest path between nodes in drug network A, $\langle d_{BB}\rangle$ represents the shortest path between nodes in drug network B, and $\langle d_{AB}\rangle$ represents the shortest path between nodes in drug networks A and B. When $S_{AB}<0$, the targets of the two drugs are located in distinct but complementary regions within the PPI network. This indicates that, while both drugs are relevant to the same disease or biological process, they act on separate signaling pathways or molecular mechanisms. Biologically, such complementary targeting broadens therapeutic coverage by modulating different disease components. This often results in synergistic effects, where the combined impact of the two drugs exceeds their individual effects. Such synergy typically occurs when drugs act on different stages of disease progression or influence parallel pathways, ultimately enhancing therapeutic efficacy and reducing the likelihood of drug resistance.

We believe that a drug combination has a therapeutic effect only when it has a specific relationship with the disease module as captured by the complementary exposure pattern in the target module of the two drugs [35]. $S_{AB}\geq 0$ usually means that two drug target modules in the network are isolated from each other, while $S_{AB}<0$ indicates that the two drug target modules overlap with each other.

### 2.6. Driver Gene Identification Based on Network Proximity Indices

The shortest distance is the sum of the distances between all nodes in A and B, and then normalized by the product of their sizes [36]:

$$d_{I(A)I(B)}^{shortest}=\frac{1}{\left\|I(A)\right\|\times \left\|I(B)\right\|}\sum_{a\in I\left(A\right),b\in I(B)}di(a,b)$$

where *I (A)* = (*a*1, *a*2, *a*3, …) is the set of components in module a and *di(a, b)* is the distance between two component nodes in modules *a* and *b*. The shortest distance measures the relationship between two functional modules in a biological network. It represents the average shortest path length between two gene modules, reflecting their proximity and directness of interaction. If the shortest path between two modules is shorter, they will likely interact through fewer intermediary genes, indicating that they will likely be functionally closer together.

Flow centrality (FC value) measures the ability of a gene to facilitate the transfer of information between different functional modules. In biological networks, certain genes or proteins serve as critical ’bridges’ connecting multiple signaling pathways or regulatory modules, enabling the coordination of diverse biological processes. Genes with high flow centrality are often at the crossroads of multiple pathways and play a central role in network regulation. Targeting these genes can influence multiple biological pathways simultaneously, potentially enhancing therapeutic efficacy. The flow centrality of node v is defined as [37]:

$${FC}^{A,B}\left(v\right)=\frac{1}{\left|A\right|\left|B\right|}\sum_{a\in A,b\in B}\frac{\sigma_{ab}(v)}{\sigma_{ab}}$$

For *A* and *B*, $\sigma_{ab}(v)$ is the number of shortest paths from *a* to *b* passing through node *v*, $\sigma_{ab}$ is the total number of shortest paths from *a* to *b*, |A| is the size of *A*, |B| is the size of *B*. Modules *A* and *B* exchange roles in undirected networks.

By integrating network proximity indices, including $d_{I(A)I(B)}^{shortest}$ and FC, we can systematically evaluate and identify driver genes in biological networks. The shortest path distance quantifies the network proximity between gene modules, capturing their functional relevance, while FC highlights genes that serve as critical mediators of information transfer between modules. By prioritizing genes with smaller $d_{I(A)I(B)}^{shortest}$ and higher FC, we can identify those that are both functionally interconnected and topologically influential, thereby effectively uncovering driver genes.

### 2.7. Functional Annotation Gene Analysis

The Database for Annotation, Visualization and Integrated Discovery (DAVID) (https://david.ncifcrf.gov/, accessed on 7 April 2023) was used for Gene Ontology (GO) analysis and Kyoto Encyclopedia of Genes and Genomes (KEGG) pathway enrichment analyses to characterize the function of the Sy_module. A *p*-value < 0.05 was considered statistically significant using *Homo sapiens* as a GO item and for KEGG pathway analysis. Weishengxin (https://www.bioinformatics.com.cn/, accessed on 7 April 2023) was used to draw a genetic enrichment diagram and a pathway mechanism map was created using the KEGG Mapper platform (https://www.genome.jp/kegg/mapper/, accessed on 7 April 2023).

### 2.8. Animal Model and Drug Administration

To validate the effectiveness of the DiMS strategy and the JU Sy_modules, in vivo animal experiments were conducted to investigate the expression patterns of driver-gene-encoded proteins and pathway proteins across each experimental group. The animal experiments were approved by the Ethics Committee of the Animal Laboratory of the Institute of Chinese Materia Medica (license number: 2023B047) and adhered to the ethical guidelines of the National Institutes of Health Guide for the Care and Use of Laboratory Animals.

Forty male Sprague Dawley rats, aged 6 weeks and weighing approximately 200 g, were provided by Beijing Vital River Laboratory Animal Technology Co., Ltd. (Beijing, China). The selection of SD rats was due to our previous study [38], which aimed to validate the driver proteins across species and enhance the validity of the analysis results. After one week of adaptive feeding, the animals were divided into five groups (six rats per group): sham, vehicle, JA, UA, and JU groups. Rats were anesthetized with 2% pentobarbital (4 mg/kg, intraperitoneally) before undergoing surgical procedures to induce MCAO. An intraluminal filament was inserted through the internal carotid artery to occlude the left middle cerebral artery (MCA) at its origin for 90 min. After this period, the filament was carefully removed to restore blood flow, initiating a 24 h reperfusion period to induce ischemia–reperfusion injury. Animals in the sham-operated group underwent the same surgical maneuver, but no wire plug was inserted. Immediately after the filament was removed, drugs were administered via tail vein injection at a dose of 2 mL/kg. After 24 h, the animals were anesthetized via intraperitoneal injection of 2% pentobarbital (4 mg/kg), followed by blood collection to induce euthanasia. Subsequently, brain tissue was harvested for further analysis. The total duration of the experimental protocol is approximately 9 days. In this study, we used three rats per group to observe the previously confirmed effect of JU in reducing cerebral infarct volume. For Western blot analysis, three rats per group were allocated (total of fifteen rats for WB analysis). During the experiment, a heating pad maintained rectal temperature at 37.0–37.5 °C, and an infrared lamp kept the body temperature at 37 °C during surgery. According to the infarct volume of these rats, we can determine whether the operation is successful. The post-operative mortality rate was approximately 10%, and two additional rats were kept as backups. Blinding procedures were implemented throughout the study. The experimenter was unaware of the animal’s group allocation during drug administration, behavioral testing, and data collection.

Previous studies revealed the LD50 of JA and UA were 3 g/kg and 10 g/kg [39,40]. The doses for subsequent experiments were selected based on their demonstrated neuroprotective effects in ischemic stroke models [29,38]. The selected doses were JA (25 mg/mL), UA (7 mg/mL), and JU (a 1:1 combination of JA and UA), along with sham (0.9% NaCl) and vehicle (post-MCAO/R + 0.9% NaCl). Drugs were administered via tail vein injection at a dose of 2 mL/kg, resulting in the following drug dosages per kilogram: JA (50 mg/kg), UA (14 mg/kg), and the JU combination (25 mg/kg of JA and 7 mg/kg of UA). The administration was performed immediately after releasing the ligation. JA at 25 mg/mL significantly improved neurological function and reduced infarct volume, while UA at 7 mg/mL also exhibited protective effects. The JU combination was used to investigate potential synergistic neuroprotection following ischemic injury [41].

### 2.9. Evaluation of the Neurological Deficit Score and Infarct Volume

After 90 min of ischemia, the neurological deficits were assessed using Longa’s scoring method in each group (*n* = 4) [42]. The Longa score uses the following criteria: 0 points, no neurological deficit (similar to sham group); 1 point, failure to extend the left forepaw, indicating slight neurological deficit; 2 points, circling to the left, suggesting neurological deficit; 3 points, difficulty walking and hemiplegic behavior, showing severe neurological deficit; 4 points, inability to walk freely and significantly decreased level of consciousness.

At 24 h post-ischemia, rats were randomly selected from each group for calculating the cerebral infarction volumes via 2,3,5-triphenyltetrazolium chloride (TTC) staining, and the infarct size was measured by ImageJ software (version 1.53).

### 2.10. Western Blotting

After cell lysis and protein extraction, a standard Western blot (WB) experiment was conducted: 40 mg of total tissue protein was separated by 10% sodium dodecyl sulfate–polyacrylamide gel electrophoresis (SDS-PAGE) gel and transferred to a nitrocellulose membrane. Subsequently, the PVDF membrane was immersed in a blocking solution (TBST) containing 5% skimmed milk powder and blocked at room temperature for 2 h. The PVDF membrane was then immersed in the primary antibody incubation solution and kept overnight at 4 °C. After removing the excess primary antibody through washing, the PVDF membrane was further incubated in the secondary antibody solution at room temperature for 2 h. The secondary antibodies used were: anti-NRF1 (diluted 1:10,000, Proteintech, 12482-1-AP, RRID:AB_2282876), anti-CUL3 (diluted 1:10,000, Affinity, DF6223, RRID:AB_2838189), anti-ITGA4 (diluted 1:10,000, Proteintech, 19676-1-AP, RRID:AB_10640907), and anti-FN1 (diluted 1:10,000, Proteintech, 66042-1-IG, RRID:AB_11182385). β-actin (diluted 1:20,000, Affinity, T0022, RRID:AB_2839417) was employed as an internal control for comparison. The membrane was air-dried and scanned, and the grayscale intensity of the bands was analyzed using Image-Pro Plus 6.0 software to quantify the protein band densities.

### 2.11. Literature Validation

All relevant literature on Web of Science, Google Scholar, PubMed, and China National Knowledge Infrastructure were searched to verify the gene function pathways of the selected driver genes. The keyword ’cerebral ischemia’ and the selected driver genes were entered into the search bar, and the literature was read and judged to find a supporting verification text.

### 2.12. Statistical Analysis

Data are expressed as dot plots overlaid with bar group means ± S.D. Statistical analyses were performed using GraphPad Prism version 8.0.2 (GraphPad Software). For comparisons between two groups, the Mann–Whitney U test was used, while the Kruskal–Wallis test followed by Dunn’s post hoc test was applied for multiple group comparisons. Results were considered statistically significant at *p* < 0.05. The statistical analyst was blinded to the group allocation during data analysis, and group information was revealed only after the statistical analysis was completed.

## 3. Results

### 3.1. Co-Expression Modules of Three Drug Groups

Our previous study found that JA, UA, and JU effectively reduce ischemic infarct volume compared with the sham group, and JU is superior to the single drug groups JA and UA [43]. This proves the synergistic effect of JU in the treatment of CI.

**Figure 1:**
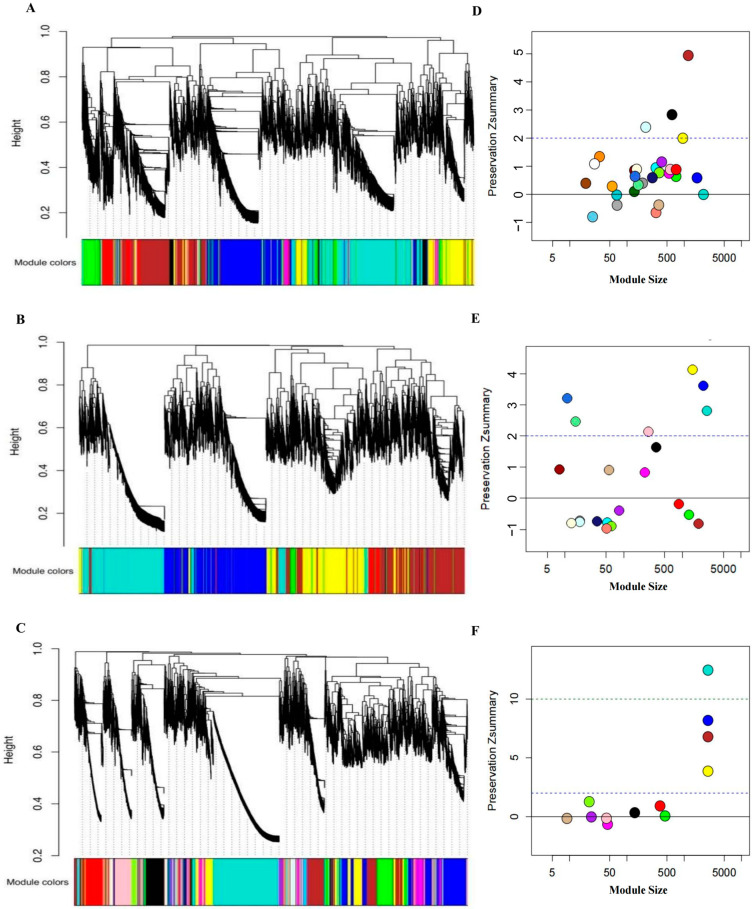
Hierarchical clustering tree diagram of three drug groups and hierarchical clustering tree diagram of On_modules. (**A**–**C**) Hierarchical clustering tree diagram of three drug groups and hierarchical clustering tree diagram of On_modules (JA, UA, and JU). (**D**–**F**) On_modules of JA, UA, and JU compared with sham group. JA, UA, and JU modules are labeled with a specific color. Modules are color-coded, with each color representing a distinct gene co-expression module without specific biological meaning. JA, jasminoidin; JU, combination of jasminoidin and ursodeoxycholic acid; UA, ursodeoxycholic acid.

A weighted gene co-expression grid map was constructed using the WGCNA package to identify the modules of the different drugs based on the expression profiles of the genes in JA, UA, and JU groups. We identified 29, 21, and 12 co-expression modules for JA, UA, and JU, respectively. Each module corresponded to a subset of the generated clusters and was assigned a unique color corresponding to a cluster (Figure 1A–C).

### 3.2. The Conserved and On_Modules of JA, UA, and JU

The biological modules in the obtained co-expression network were analyzed. The $Z_{summary}$ value in WGCNA was retained compared to the sham group, where $Z_{summary}\geq 2$ was regarded as a conserved module, and $Z_{summary}<0$ was regarded as an On_module (Figure 1D–F). All modules in the three drug groups were compared with those in the sham group, and we obtained 6 (JA- Mod-6, 9, 24, 25, 26, and 27), 11 (UA-Mod-3, 4, 6, 7, 8, 9, 11, 13, 15, 16, and 18), and 4 (JU-Mod-6, 7, 8, and 10) On_modules of JA, UA, and JU, respectively. In contrast, JA, UA, and JU contained three (JA-Mod-1, 3, and 13), six (UA-Mod-2, 10, 14, 17, 20, and 21), and four (JU-Mod-2, 3, 11, and 12) conserved modules compared to the sham group, respectively.

### 3.3. The Synergistic Modules of JU

**Figure 2:**
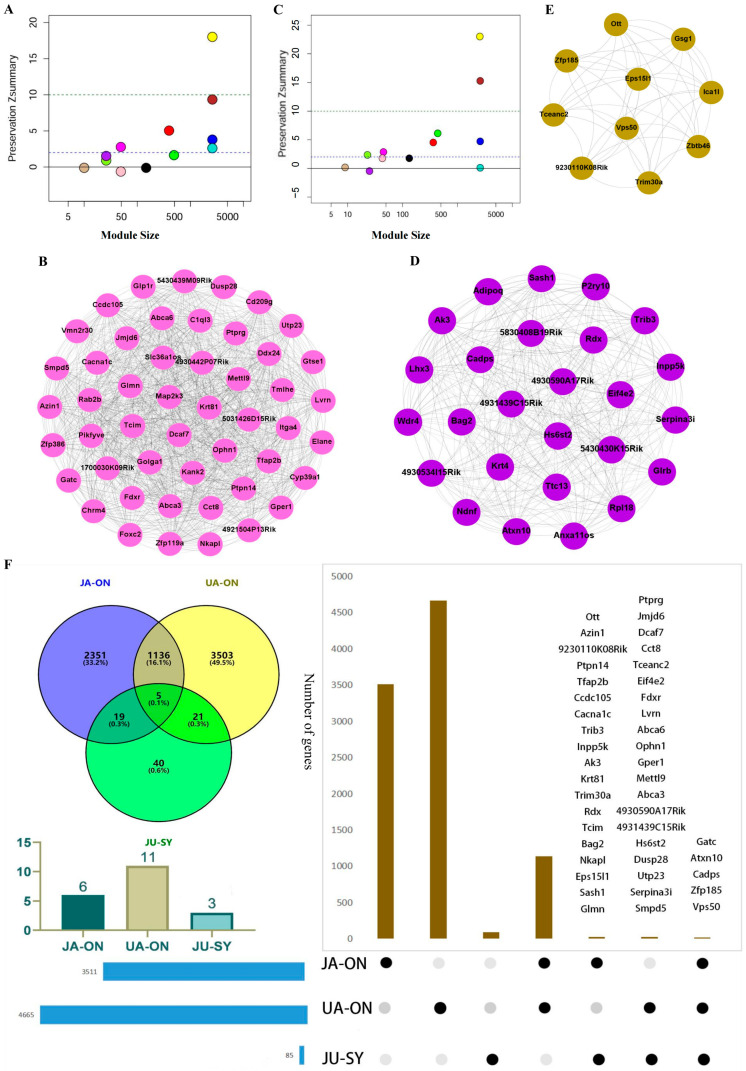
Preservation analysis and visualization of Sy_modules in JU compared to single-drug treatments (JA or UA). (**A**) Preservation Zsummary values for different gene modules in JU compared to JA. The solid line represents the threshold of 0, and the dashed line represents the threshold of 2. Modules below the solid line are considered Sy_modules (response modules), indicating significant changes in gene expression patterns under JU treatment compared to JA treatment. (**B**) Visualization of the Sy_module (JU-Mod-8) identified in JU compared to JA. (**C**) Preservation Zsummary values for different gene modules in JU compared to UA. Modules below the solid line are considered Sy_modules (response modules), indicating significant changes in gene expression patterns under JU treatment compared to UA treatment. (**D**) Visualization of the Sy_module (JU-Mod-10) identified in JU compared to UA. (**E**) Visualization of the Sy_module (JU-Mod-7) identified in JU compared to UA. (**F**) Gene overlap and information among JA On_ modules (response modules identified in JA compared to the sham group), UA On_modules (response modules identified in UA compared to the sham group), and JU Sy_modules (response modules identified in JU compared to single-drug groups). JA, jasminoidin; JU, combination of jasminoidin and ursodeoxycholic acid; UA, ursodeoxycholic acid.

Consistent with the method described in the previous step, the modules of JU were compared with those of JA and UA to identify the synergistic modules (Sy_modules) that may represent the combination therapy mechanism of JU. A Sy_module (JU-Mod-8) was obtained compared to JA (Figure 2A,B). Three Sy_modules (JU-Mod-1, 7, and 10) were obtained compared to UA (Figure 2C–E). The modules reproduced in the On_modules of JU were considered to be Sy_modules, and three Sy_modules were obtained (JU-Mod-7, 8, and 10) that were composed of 49, 26, and 10 genes, respectively. In addition, there were eight conserved modules (JU-Mod-2, 3, 4, 5, 6, 9, 11, and 12) of JU containing 3534, 3517, 491, 25, 49, 397, 3569, and 2872 genes, respectively.

The gene numbers of the On_modules of JA and UA were 3511 and 4665, respectively, and the Sy_modules had 85 genes. Among them, 1136 genes were shared by the JA On_modules and UA On_modules, 21 genes were shared by the On_modules of the UA and Sy_modules, and 19 genes were shared by the On_modules of the JA and Sy_modules. Five common genes (*Gatc*, *Atxn10*, *Cadps*, *Zfp185*, and *Vps50*) were identified in all three groups, suggesting their core involvement in post-ischemic molecular responses. Gene overlaps and information on the JA On_modules, UA On_modules, and JU Sy_modules are shown in Figure 2F. *Vps50*, a component of the endosome-associated recycling protein complex, is implicated in vesicle trafficking and intracellular transport, which are essential for neuronal homeostasis and recovery [44]. *Atxn10* is involved in RNA metabolism and gene expression regulation, processes crucial for cellular adaptation and repair following ischemic injury [45]. *Cadps* facilitates neurotransmitter release [46], while *Zfp185*, a zinc-finger protein, may function as a transcriptional regulator in response to cellular stress. *Gatc* is associated with chromatin remodeling and transcriptional regulation, playing a role in gene expression changes under ischemic conditions [47]. Notably, these genes were consistently present in JA On_modules, UA On_modules, and JU Sy_modules, suggesting their fundamental role across different treatment conditions. Their presence in JA and UA modules may indicate involvement in distinct neuroprotective pathways activated by individual treatments, while their inclusion in the JU Sy_modules suggests a potential role in mediating the synergistic effects observed with the combined treatment.

### 3.4. The Overlap of On_Module Genes and Differentially Expressed Genes

The cDNA expression profile of 16,463 genes identified 229, 165, and 225 DEGs in JA, UA, and JU, respectively (Supplementary Figure S1A–C). The total number of DEGs among the three drug groups was six (1.1%). There were 22 (4%) overlapping DEGs between JA and UA, 16 (2.9%) between UA and JU, and 19 (3.4%) between JA and JU (Supplementary Figure S1D). The low DEG overlap suggests that these treatments regulate distinct biological processes. JA-associated DEGs are primarily enriched in neural and immune-related pathways (e.g., glutamatergic and dopaminergic synapses, chemokine signaling), UA-associated DEGs predominantly involve inflammatory and metabolic pathways (e.g., TNF signaling, non-alcoholic fatty liver disease), while JU-associated DEGs regulate cellular stress and signal transduction (e.g., MAPK and calcium signaling). This indicates that different treatments elicit distinct molecular responses, contributing to the observed low DEG overlap. Despite this, each group’s DEGs are functionally relevant within their respective regulatory networks, potentially influencing neuroimmune interactions, inflammation, and metabolic homeostasis.

The genes in the modules or Sy_modules partially overlapped with the DEGs of each drug group. The number of overlapping genes between the JA-DEGs and the JA On_module was 42, while that between the UA-DEGs and the UA On_module was 29 (Supplementary Figure S1E), and 1 gene (*Zfp119a)* overlapped between the JU-DEGs and Sy_modules (Supplementary Figure S1F).

### 3.5. Network Synergistic Map Between Different Levels of Modules

**Figure 3:**
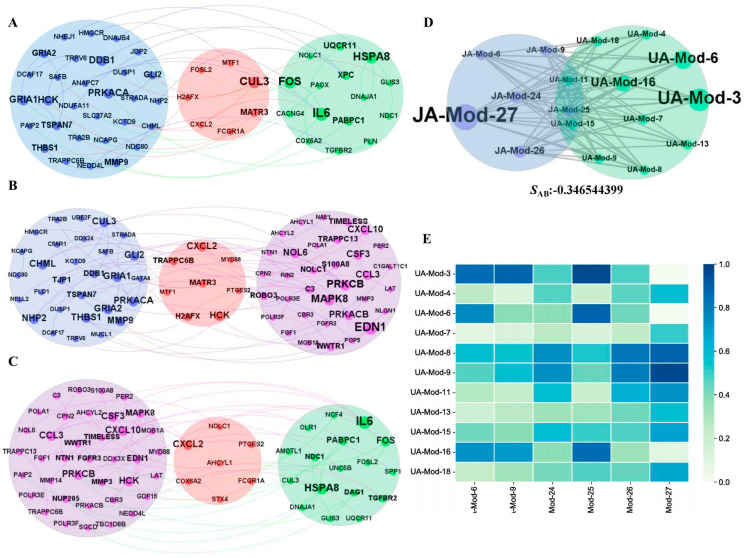
The network map of three drug groups at the DEG level and On_module level. (**A**) The network map of JA-UA at the DEG level. (**B**) The network map of JA-JU at the DEG level. (**C**) The network map of UA-JU at the DEG level. The blue module represents the JA group, the green module represents the UA, the purple module represents the JU, and the pink region represents the common gene group. (**D**) $S_{AB}$ diagram of JA On_modules and UA On_modules. (**E**) Heatmap between small modules in JA On_modules and UA On_modules. DEG, differentially expressed gene; JA, jasminoidin; JU, combination of jasminoidin and ursodeoxycholic acid; UA, ursodeoxycholic acid.

$S_{AB}$ was used to measure the synergistic relationship between different drug groups. A synergistic effect was more likely at a smaller $S_{AB}$. All $S_{AB}$ of the three drug groups were <0, and the $S_{AB}$ between JA and UA was the smallest from the perspective of DEGs (Table 1). The DEG network diagram of the three drug groups is shown in Figure 3A–C. The $S_{AB}$ values of the JA On_module and UA On_module were <0 from the perspective of On_modules. Based on $S_{AB}$, the synergistic map of On_modules between JA and UA is shown in Figure 3D,E. The synergistic relationship between both DEGs and the On_module level suggested a synergistic effect between JA and UA.

### 3.6. Identification of Driver Genes and Literature Verification

The hub genes between the On_modules of the single-agent group were calculated based on two important indicators (shortest path and FC) in the network. The top ten genes with the shortest path values and largest FC value were selected. Eight common genes (*UBC*, *APP*, *NRF1*, *JUN*, *ELAVL1*, *GRB2*, *CUL3* and *SUMO2*) and four specific genes (*YWHAZ*, *TP53*, *FN1* and *PRKACA*) were identified (Table 2). The driver gene *UBC* was not confirmed to be closely related to cerebral ischemia; however, other driver genes were identified. Many studies confirmed that *SUMO* [48,49,50], *NRF1* [51,52], *APP* [53], *JUN* [54], *ELAVL1* [55,56,57], *GRB2* [58], *CUL3* [59], *YWHAZ* [60], *TP53* [61], *FN1* [62] and *PRKACA* [63] are important targets for the treatment of CI. This proves that using the concept of $S_{AB}$ and the two indicators of shortest path and FC value may effectively screen the targets of drug combinations.

Among the identified genes, *CUL3* may exacerbate neuronal apoptosis and aggravated cerebral ischemia–reperfusion injury by negatively regulating the Nrf2 signaling pathway [59]. *GRB2* plays a key role in neuroprotection, reducing neuronal damage through the regulation of autophagy and the Akt/mTOR pathway in ischemic stroke [58]. *FN1* has been implicated in ischemic stroke by promoting inflammation, participating in repair mechanisms, and regulating cell functions in the bone marrow microenvironment [64]. Additionally, *ITGA4*, present in JU Sy_modules, may regulate cell survival and apoptosis by influencing cell adhesion and migration, ultimately affecting neuronal injury and repair [65].

### 3.7. Significant Biological Functions

We conducted GO analysis and KEGG pathway enrichment analysis using the DAVID database (https://david.ncifcrf.gov/, accessed on 15 April 2023).

**Figure 4:**
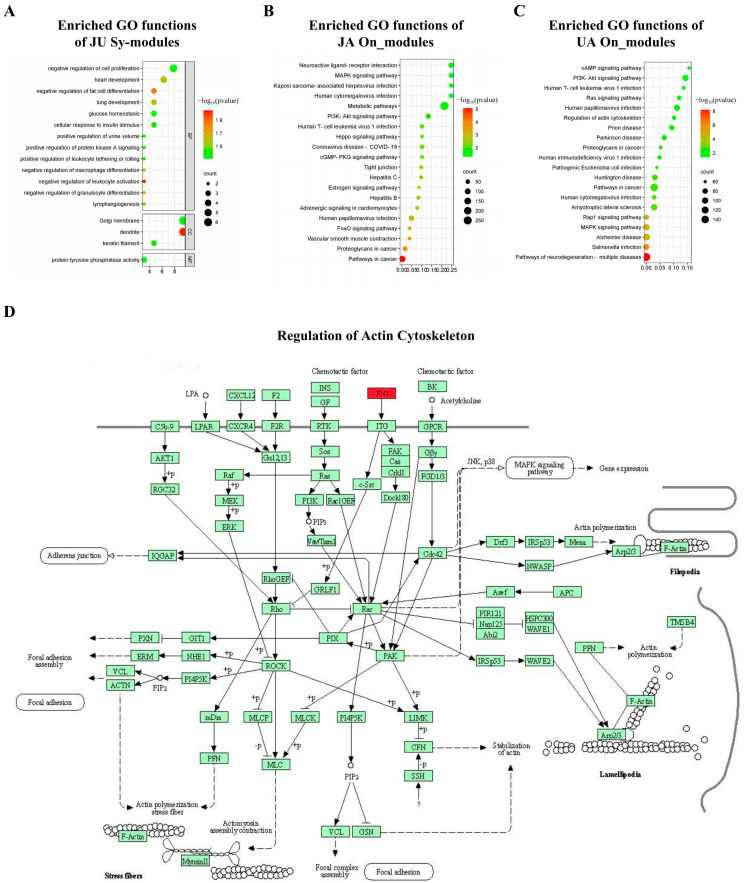
Functional enrichment of JU Sy_modules, JA On_modules, and UA On_modules. (**A**) Significant Gene Ontology (GO) terms enriched in Sy_modules, illustrating the enrichment of biological processes, molecular functions, and cellular components associated with Sy_modules, which represent response modules of JU compared to single-drug groups. (**B**) Significant Gene Ontology (GO) terms enriched in JA On_modules, illustrating the biological processes, molecular functions, and cellular components associated with JA On_modules, which represent response modules of JA compared to the sham group. (**C**) Significant Gene Ontology (GO) terms enriched in UA On_modules, illustrating the biological processes, molecular functions, and cellular components associated with UA On_modules, which represent response modules of UA compared to the sham group. (**D**) Sy_modules enriched in the regulation of the actin cytoskeleton signaling pathway, with the driver genes of the JU Sy_module (highlighted in red in the figure) showing strong associations with this pathway, suggesting that JU may influence cytoskeletal dynamics, which could play a role in cellular structural remodeling. JA, jasminoidin; JU, combination of jasminoidin and ursodeoxycholic acid; UA, ursodeoxycholic acid.

In biological process (BP), cellular component (CC), and molecular function (MF) of GO enrichment analysis categories, the significantly enriched functions of JU Sy_modules were identified (*p* < 0.05, Figure 4A): the BP functions were mainly related to regulation of development and maturation, regulation of protein kinase A signaling, and cell proliferation regulation, among others; the CC functions were mainly related to Golgi membranes and dendrites; the MF functions were related to protein tyrosine phosphatase activity.

For JA and UA On_modules (Figure 4B,C), the enriched KEGG pathways mainly involved the mitogen-activated protein kinase (MAPK), phosphoinositide-3 kinase-protein kinase B (PI3K-Akt), and cyclic adenosine monophosphate (cAMP) signaling pathways. For JU Sy_modules, KEGG enrichment analysis identified a specific enriched pathway, i.e., regulation of the actin cytoskeleton (*p* = 0.038, Figure 4D). In this regulation pathway, *FN1* acted directly on *ITG*, promoting dendritic growth, providing neuroprotective effects, and improving blood–brain barrier permeability [62].

### 3.8. Protein Expression of Driver Genes and JU Sy_Modules’ Pathway

To validate the essential roles of the DiMS strategy and the JU Sy_modules in contributing to the synergistic effect of JU, WB analysis was used to examine the expression patterns of driver proteins and pathway proteins across different groups.

In our experiment, the TTC staining showed an increased infarct volume in the vehicle group compared to the sham group, and the infarct volumes were decreased in all of the three drug groups compared to that in the vehicle group (*n* = 3, Supplementary Figure S2A,B).

**Figure 5:**
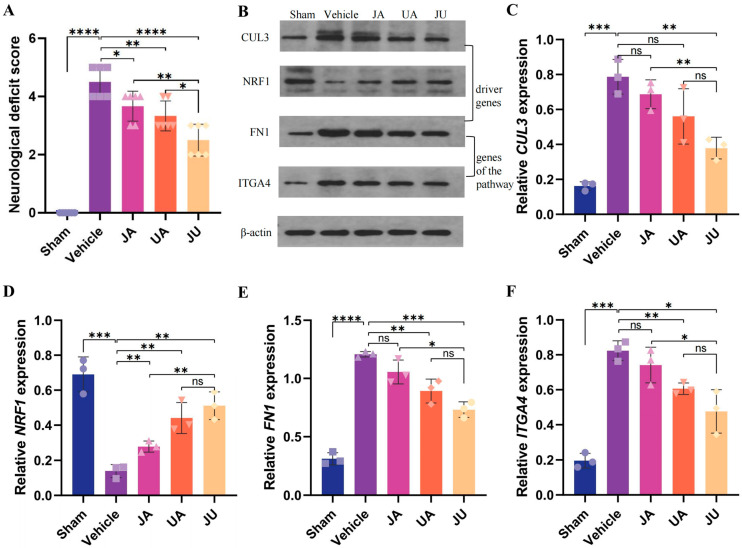
Neurological deficit scores and validation of driver gene and Sy_module gene expression levels at 24 h post-MCAO/R. (**A**) Comparison of the neurological deficit scores across each group 24 h after MCAO/R (*n* = 6). This measure provides insight into the functional impairment following ischemic injury, with the score reflecting the severity of the neurological deficits. A lower score indicates milder neurological dysfunction. (**B**) Western blotting results showing the expression levels of three representative proteins, with β-actin as a loading control. Differences in band intensity indicate variations in protein expression across treatment groups, reflecting the molecular effects of JU, JA, and UA treatments on these proteins. (**C**–**F**) Validation of the expression levels of key driver genes (*CUL3*, *NRF1*) and pathway-related proteins (*FN1*, *ITGA4*) in each group (*n* = 3), as determined by Western blotting. The comparison of protein expression levels among the groups reflects the differential regulation of these molecules after JU, JA, or UA treatment, compared to the sham or single-drug groups. * *p* < 0.05, ** *p*< 0.01, *** *p* < 0.001, **** *p* < 0.0001, and ns, non-significant.

Additionally, compared with that of the sham group, the neurological deficit score of the vehicle group (4.5, *p* < 0.0001) was increased. Compared to that of the vehicle group, those of the UA (3.25, *p* < 0.05) and JU (2.5, *p* < 0.01) groups were decreased (Figure 5A). Notably, compared to JA, JU (2.5, *p* < 0.05) was more effective at improving neurological deficits.

The expression levels of three driver genes (*CUL3*, *NRF1*, *FN1*) identified through the DiMS strategy were analyzed via WB (Figure 5B–E). The driver genes *FN1* and *ITGA4* in the JU Sy_modules are located within the pathway that regulates the actin cytoskeleton (Figure 5E,F).

Figure 5C presents the relative expression levels of *CUL3* across different treatment groups. Compared to the vehicle group (FC = 1), the sham group showed a significant decrease in *CUL3* expression (FC = 0.21, *p* < 0.001). The JA group exhibited a moderate reduction (FC = 0.87), and the UA group showed a slight decrease (FC = 0.71), though neither change was statistically significant. In contrast, the JU group exhibited a further significant reduction in *CUL3* expression (FC = 0.48, *p* < 0.01), indicating a statistically significant downregulation in response to treatment. Additionally, *CUL3* expression in the JU group was significantly lower than that in the JA group (*p* < 0.01), suggesting that JU treatment induces a more pronounced downregulation of *CUL3* expression.

Figure 5D presents the relative expression levels of *NRF1* across different treatment groups. Compared to the vehicle group (FC = 1), the sham group exhibited a significant increase in *NRF1* expression (FC = 5.00, *p* < 0.001). The JA group showed a moderate increase (FC = 2.00, *p* < 0.01), and similarly, the UA group demonstrated a slight increase (FC = 3.20, *p* < 0.01). In contrast, the JU group exhibited a further increase in *NRF1* expression (FC = 3.70, *p* < 0.01), suggesting a more pronounced upregulation. Additionally, *NRF1* expression in the JU group was significantly higher than in the JA group (*p* < 0.01), indicating that JU treatment is more effective in upregulating *NRF1* expression compared to JA.

Figure 5E illustrates the relative expression levels of *FN1* across different treatment groups. Compared to the vehicle group (FC = 1), the sham group showed a significant decrease in *FN1* expression (FC = 0.26, *p* < 0.001). The UA group exhibited a moderate but significant reduction (FC = 0.74, *p* < 0.01), while the JU group showed a further decrease (FC = 0.61, *p* < 0.001). The JA group exhibited a slight reduction (FC = 0.87), but this change was not statistically significant. Further comparisons revealed that *FN1* expression in the JU group was significantly lower than in the JA group (*p* < 0.05), indicating a stronger downregulation effect induced by JU treatment.

Figure 5F presents the relative *ITGA4* expression levels across different treatment groups. Compared to the vehicle group (FC = 1), the sham group exhibited a significant decrease in *ITGA4* expression (FC = 0.24, *p* < 0.001). The JA group showed a moderate increase (FC = 0.90), but this change was not statistically significant. The UA group demonstrated a slight increase (FC = 0.74, *p* < 0.01). In contrast, the JU group displayed a further reduction in *ITGA4* expression (FC = 0.58, *p* < 0.05), indicating a statistically significant downregulation in response to treatment. Moreover, *ITGA4* expression in the JU group was significantly lower than in the JA group (*p* < 0.05), suggesting that JU treatment induces a more pronounced downregulation of *ITGA4* expression compared to JA.

The driver genes *FN1* and *ITGA4* in the JU Sy_modules are located within the pathway that regulates the actin cytoskeleton. WB results (Figure 5E,F) revealed significant downregulation of *FN1* (*p* < 0.001) and *ITGA4* (*p* < 0.05) in the JU group compared to the vehicle group.

## 4. Discussion

Prescriptions usually play a role in the formation of multi-components, multi-targets, and multi-pathways owing to the polypathogenic genes of complex diseases such as CI. However, it is challenging to fully explain the synergistic mechanism of drug combination therapy [66,67]. A variety of genes, proteins, and other cellular components constitute a complex network in complex life activities such as diseases and drug intervention. Increasing evidence shows that drug etiology and mechanisms of action are modular. The modular network of drugs modulates diseases to produce interventions during drug intervention [68,69,70]. Drugs intervene in diseases through a network, wherein the neighbors of the response gene usually have similar intervention responses, and some genes and neighbors have the same intervention response. Modules are considered gene, protein, or metabolic clusters that are functionally related or respond together when drugs intervene in a disease at the molecular level. These clusters are gathered to drive the modular process of disease drug treatment. This study systematically identified three synergistic modules and twelve driving genes of JU based on the gene expression data of JA, UA, and JU in the anti-CI model through a network topologic method and modular analysis of complex networks. Furthermore, the synergistic mechanism of JU was revealed in the treatment of CI from the field of modular networks and systems. The modular targeted research strategy was improved to provide a new perspective for exploring the advantages of drug combination therapy and screening the synergistic mechanisms of complex diseases.

The specific pathway of the JU Sy_modules was the regulation of the actin cytoskeleton based on the KEGG pathway analysis (Figure 4D). Dynamic changes in the actin cytoskeleton are key to a variety of cellular activities, including cell movement and phagocytosis [71], and the dysfunction of cytoskeletal proteins can lead to various diseases. Actin exists as monomeric and filamentous forms in cells and the transition between these two forms results in a steady-state equilibrium [72]. Rho mediates the regulation of the endothelial actin cytoskeleton, upregulates eNOS expression, and improves severe ischemia after middle cerebral artery occlusion [73]. Responses to GO terms such as stem dendrites, protein tyrosine phosphatase, and cell proliferation are closely related to protective effects against cerebral ischemia [74,75,76]. Regulation of the actin cytoskeleton is closely related to CI, such as neuroprotective effects [77,78], reduction of brain tissue damage [79], and changes in blood–brain barrier permeability [80]. We concluded that the identified Sy_modules are closely related to CI. In this study, we identified Sy_modules that are significantly enriched in the regulation of the actin cytoskeleton pathway, highlighting the potential relevance of this pathway in the treatment of CI. Following ischemic injury, disruption of the neuronal cytoskeleton leads to impaired morphology and function. Dynamic remodeling of the cytoskeleton plays a critical role in neural repair, particularly in neuronal migration, synaptic reconstruction, and neural network recovery. Targeted modulation of cytoskeletal proteins can aid in the recovery of neuronal morphology and promote the restoration of neurological function. Furthermore, the enrichment of Sy_modules in the regulation of the actin cytoskeleton pathway suggests that multi-target drugs may enhance therapeutic effects through synergistic actions. This mechanism provides a new perspective for the development of targeted therapies for CI and promotes the clinical translation of potential drug therapies. Following the analysis of the synergistic mechanism of JU and the involvement of the actin cytoskeleton pathway, it is important to highlight the potential correlation between the observed protein expression changes and behavioral improvements, particularly in the JU group. Based on the experimental results, there may be a correlation between neurological deficit scores and protein expression changes. The JU group exhibited significant downregulation of *CUL3* and *FN1*, along with upregulation of *NRF1*, which was associated with behavioral improvements. Notably, the behavioral improvement in the JU group was more pronounced, and the changes in protein expression were more substantial, suggesting a link to neurorestoration and functional recovery. This finding reinforces the importance of multi-target interventions in enhancing therapeutic outcomes for complex diseases such as CI.

**Figure 6:**
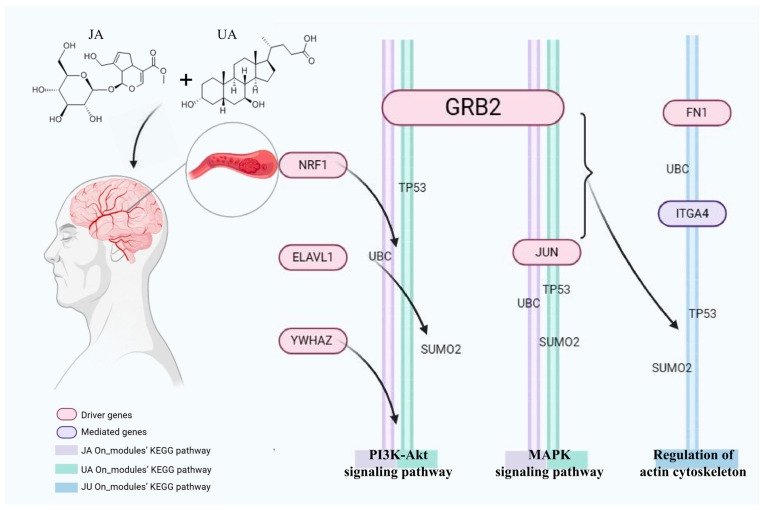
The mechanism map of cerebral ischemia in JU. The circles represent the genes and the rectangles represent the enrichment pathways. The red genes are the identified driver genes, the purple genes play mediating roles, and the driver genes drive the purple genes to complete the therapeutic effect. JU, combination of jasminoidin and ursodeoxycholic acid.

The network-topology-based driver node set in a biological network can screen hub targets of drug treatments, such as synergistic effects. When $S_{AB}<0$, overlap exists between the On_modules of JA and UA in a complex network according to the calculation results of the JA On_ modules and UA On_modules. Local disturbance of JA in cerebral ischemia may also be involved in the same way UA intervenes in diseases with common or similar pharmacological characteristics. Furthermore, 12 driver genes (*UBC*, *APP*, *NRF1*, *JUN*, *ELAVL1*, *GRB2*, *CUL3*, *SUMO2*, *YWHAZ*, *TP53*, *FN1* and *PRKACA*) were identified by calculating the shortest path and FC value. Among them, 11 driver genes were closely related to CI, as supported by the literature: *SUMO* [48,49,50], *NRF1* [51,52], *APP* [53], *JUN* [54], *ELAVL1* [55,56,57], *GRB2* [58], *CUL3* [59], *YWHAZ* [60], *TP53* [61], *FN1* [62] and *PRKACA* [63]. These results demonstrate the effectiveness of the Sy_modules, JA, and UA in elucidating the JU synergy mechanism through driving genes (Figure 6).

*FN1* promotes endothelial cell proliferation and migration through the integrin/FAK/PI3K/Akt axis, enhancing microvascular formation while regulating extracellular matrix (ECM) remodeling to maintain vascular stability. *FN1* also strengthens endothelial cell junctions, improving blood–brain barrier (BBB) function, and upregulates Bcl-2 to promote neuronal survival [62]. Additionally, *ITGA4*, as integrin α4, plays a crucial role in post-ischemic angiogenesis by facilitating endothelial cell migration and vascular formation, as well as regulating VE-cadherin and occludin to maintain BBB integrity [65,81]. *ITGA4* modulates inflammatory cell infiltration, reducing inflammation-induced damage and supporting tissue repair in ischemic regions. *FN1* and *ITGA4* may act synergistically in vascular remodeling, with *FN1* mediating ECM formation to support ITGA4-driven cell adhesion and migration. Together, they contribute to vascular repair and functional recovery in ischemic brain tissue. These mechanisms suggest that *FN1* and *ITGA4* may serve as potential therapeutic targets for ischemic stroke, providing new insights into post-ischemic vascular repair. *NRF1* plays a vital role in mitochondrial biogenesis and redox signaling during cerebral ischemia. It regulates the expression of nuclear-encoded mitochondrial genes, promoting mitochondrial biogenesis, with the help of *PGC-1α*. *NRF1* also enhances antioxidant defenses by activating genes like *SOD2*, *GPX1*, and *CAT*, which reduce oxidative stress and neuronal damage. Through its regulation of mitochondrial function and redox balance, *NRF1* provides neuroprotection in ischemic stroke, making it a potential therapeutic target [82].

The top 20 overlapping pathways were the PI3K/Akt and mitogen-activated protein kinase/extracellular regulated protein kinases (MAPK/ERK) signaling pathways according to the results of gene function enrichment analysis of JA-DEGs and UA-DEGs. The PI3K/Akt signaling pathway is considered closely related to the occurrence and development of CI and can play an important regulatory role in a series of cascade reactions (including apoptosis, autophagy, oxidative stress, and neuroinflammation) [83,84,85,86,87,88]. The MAPK/ERK signaling pathway plays a role in the inflammatory response and blood–brain barrier dysfunction and is involved in autophagy, which is closely related to CI [89,90,91,92]. This study determined that the $S_{AB}$ values of the JA-DEGs and UA-DEGs in the network were negative, and the driver genes supported by the literature were obtained using the same calculation method. These results verified the effectiveness of the strategy used to explore the combined treatment mechanism.

Although the DiMS method has advantages in module identification, it may still produce false-positive modules that do not accurately reflect the biological characteristics of the system. In future studies, we plan to validate the identified modules across different datasets or conditions and use multi-angle validation to assess their consistency.

## 5. Conclusions

In conclusion, module-based $S_{AB}$ and network topological analysis identified three Sy_modules and twelve driver genes associated with JU in CI therapy. The driver genes of *CUL3*, *NRF1*, *FN1*, *ITGA4* in Sy_modules and pathways were validated using in vivo experiments. These findings demonstrate JU can synergistically mitigate CI–reperfusion injury through regulating the driver genes inducing PI3K-Akt, MAPK, and actin cytoskeleton pathways. The findings highlight the innovative use of modular multi-target analysis to unravel the synergistic mechanisms of JU, providing a deeper understanding of its therapeutic potential. Future research should focus on further validating these pathways in clinical settings and exploring the applicability of the DiMS strategy for combination therapies in other complex diseases.

## Figures and Tables

**Table 1:** Detailed SAB values of JA-JU, JA-JU, and UA-JU at the DEG level.

Module-a	Module-a	S_AB_
JA-DEGs	UA-DEGs	−0.266
JA-DEGs	JU-DEGs	−0.183
UA-DEGs	JU-DEGs	−0.149

Abbreviations: DEG, differentially expressed gene; JA, jasminoidin; JU, combination of jasminoidin and ursodeoxycholic acid; UA, ursodeoxycholic acid.

**Table 2:** The literature support and data of JA and UA On_modules.

Driver Gene	The Shortest Distance	Flow Centrality	Literature
UBC	5904	2,442,296.72	-
SUMO2	8758	44,576.78	[48,49,50]
NRF1	8445	156,372.58	[51,52]
APP	8420	171,885.22	[53]
JUN	8589	52,966.16	[54]
ELAVL1	8601	89,125.44	[55,56,57]
GRB2	8746	61,624.5	[58]
CUL3	8757	31,379.5	[59]
YWHAZ	8828	-	[60]
TP53	8841	-	[61]
FN1	-	38,194.56	[62]
PRKACA	-	33,893.5	[63]

Abbreviations: JA, jasminoidin; UA, ursodeoxycholic acid.

## Data Availability

The original contributions presented in this study are included in the article/Supplementary Material. Further inquiries can be directed to the corresponding authors.

## Supplementary Materials

The following supporting information can be downloaded at: https://www.mdpi.com/article/10.3390/biomedicines13040938/s1, Figure S1: Differentially expressed genes (DEGs) and their overlap across treatments and modules. Figure S2: TTC Staining and Infarct Volume Analysis in Rat Models.
