# Supplementary figures and images for: Modular-Based Synergetic Mechanisms of Jasminoidin and Ursodeoxycholic Acid in Cerebral Ischemia Therapy
Source: Biomedicines. 2025 Apr 11;13(4):938. doi: 10.3390/biomedicines13040938 (PMC12025273; doi:10.3390/biomedicines13040938)

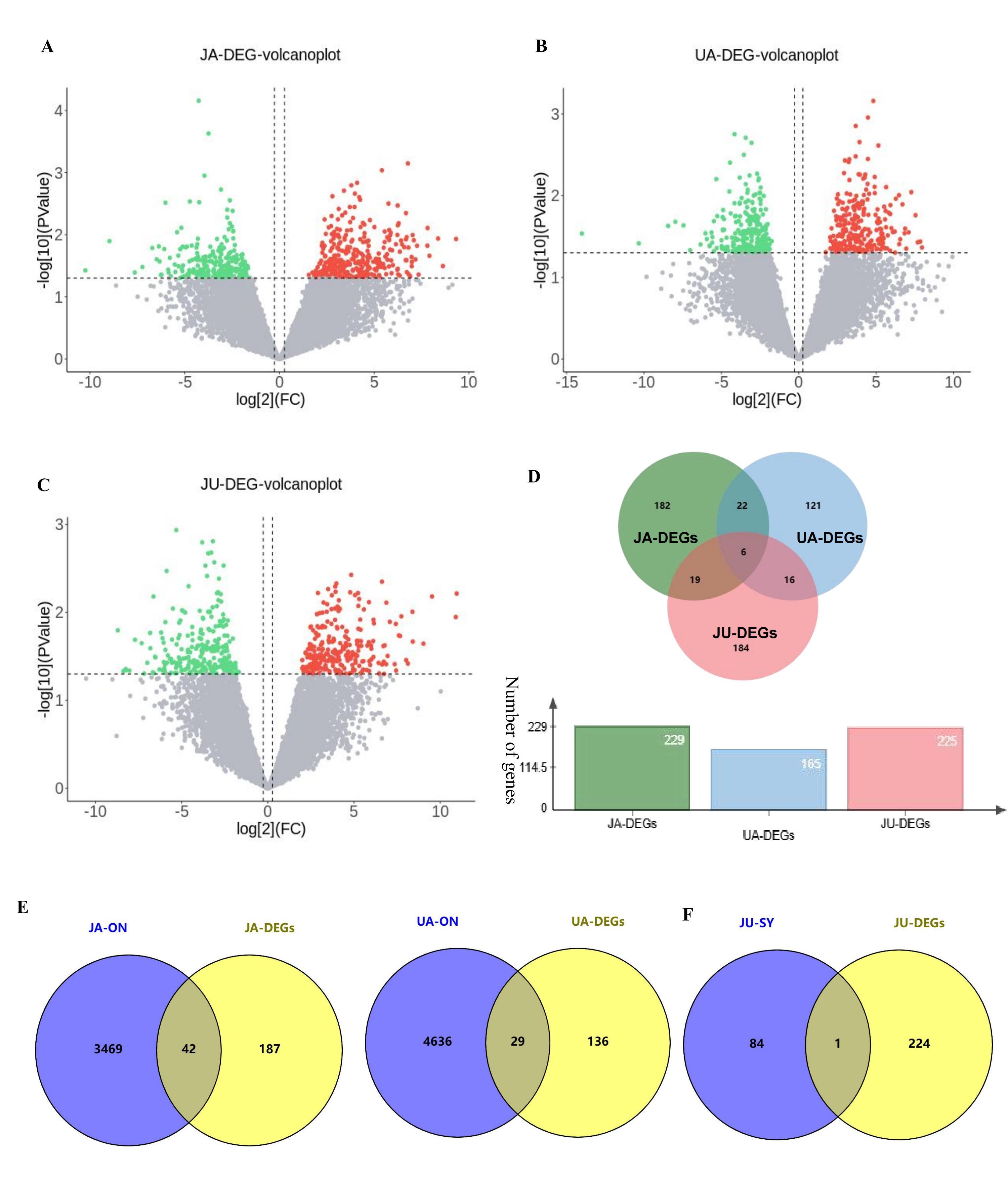

Supplement: Supplementary file 1 [file biomedicines-13-00938-s001.zip › Supplementary Figure S1.tiff]

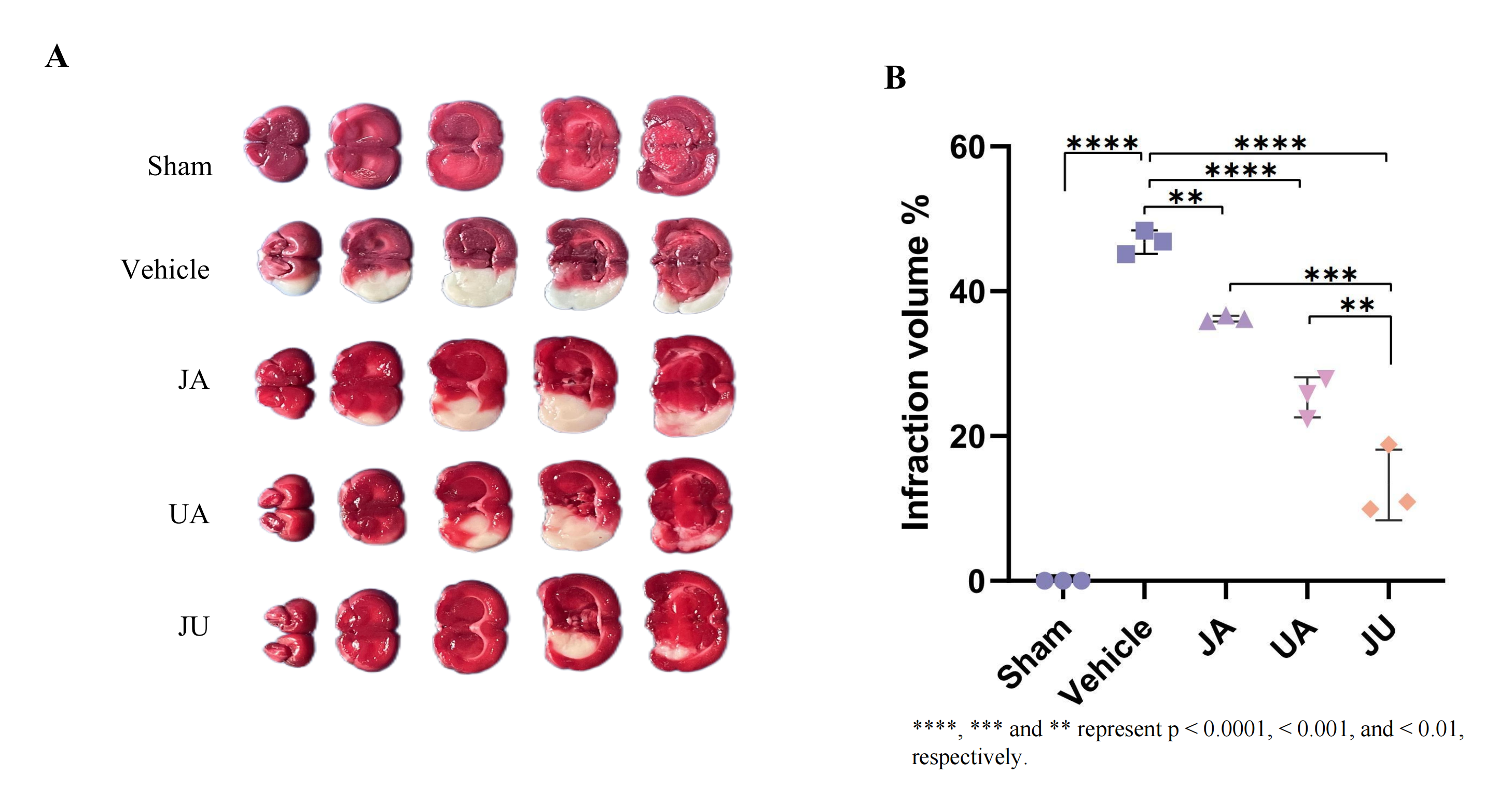

Supplement: Supplementary file 1 [file biomedicines-13-00938-s001.zip › Supplementary Figure S2.tiff]
